# Methylation of *WTH3*, a possible drug resistant gene, inhibits *p53 *regulated expression

**DOI:** 10.1186/1471-2407-8-327

**Published:** 2008-11-07

**Authors:** Kegui Tian, Yuezeng Wang, Yu Huang, Boqiao Sun, Yuxin Li, Haopeng Xu

**Affiliations:** 1 Department of Biochemistry and Cell Biology, State University of New York at Stony Brook, NY 11794, USA; 2 Laborotary of Pathobiology, Jilin University, Changchun, PR China; 3 National Engineering Laboratory for Druggable Gene and Protein Screening, Northeast Normal University, Changchun, PR China; 4 Center of Life and Food Sciences Weihenstephan, Technische Universität München, Germany

## Abstract

**Background:**

Previous results showed that over-expression of the *WTH3 *gene in MDR cells reduced *MDR1 *gene expression and converted their resistance to sensitivity to various anticancer drugs. In addition, the *WTH3 *gene promoter was hypermethylated in the MCF7/AdrR cell line and primary drug resistant breast cancer epithelial cells. *WTH3 *was also found to be directly targeted and up regulated by the *p53 *gene. Furthermore, over expression of the *WTH3 *gene promoted the apoptotic phenotype in various host cells.

**Methods:**

To further confirm *WTH3*'s drug resistant related characteristics, we recently employed the small hairpin RNA (shRNA) strategy to knockdown its expression in HEK293 cells. In addition, since the *WTH3 *promoter's p53-binding site was located in a CpG island that was targeted by methylation, we were interested in testing the possible effect this epigenetic modification had on the *p53 *transcription factor relative to *WTH3 *expression. To do so, the *in vitro *methylation method was utilized to examine the *p53 *transgene's influence on either the methylated or non-methylated *WTH3 *promoter.

**Results:**

The results generated from the gene knockdown strategy showed that reduction of *WTH3 *expression increased *MDR1 *expression and elevated resistance to Doxorubicin as compared to the original control cells. Data produced from the methylation studies demonstrated that DNA methylation adversely affected the positive impact of *p53 *on *WTH3 *promoter activity.

**Conclusion:**

Taken together, our studies provided further evidence that *WTH3 *played an important role in MDR development and revealed one of its transcription regulatory mechanisms, DNA methylation, which antagonized *p53*'s positive impact on *WTH3 *expression.

## Background

Multidrug resistance (MDR) is a fatal event encountered during cancer chemotherapy [1-7]. To better understand MDR development, we employed the Methylation Sensitive-Representational Difference Analysis (MS-RDA) technique [8-10] to study DNA hypermethylation events in a human MDR breast cancer cell line, MCF7/AdrR, and its parental line, MCF7/WT. As a result, the *WTH3 *gene was discovered. *WTH3 *gene's product is homologous to the *Rab6 *and *Rab6c *genes that encode small G proteins and belong to the *ras *super family [9-14]. Similar to the *Rab6*s, *WTH3 *is a house-keeping gene and its product is capable of binding to GTP molecules [15]. However, unlike the Rab6s that reside in the Golgi network, most of WTH3 is located in the cytoplasm and to a lesser degree in the nuclei. This disparity could be due to WTH3's lack of a cysteine at its C-terminus for geranyl-geranylation, a necessary post-translational modification for membrane attachment [16]. Previous studies found that the *WTH3 *gene was down regulated in MDR cell lines, and by introducing it back into those lines caused down regulation of *MDR1 *gene expression that reversed their MDR phenotypes to various anti-cancer drugs [9,15]. Our research revealed that hypermethylation (an epigenetic modification event in mammals, which represses gene expression) [17-22] of the *WTH3 *promoter and transcription factor modulation were involved in its differential expression in MCF7/AdrR versus MCF7/WT cells [15]. Furthermore, the hypermethylation event was also observed in primary drug resistant breast cancer cells [23].

Recently, we identified a p53-binding motif (p53M) in the *WTH3 *gene promoter, which was located in a CpG island that was targeted by DNA methylation [15,23,24]. The *p53 *gene product is a transcription factor that functions as a tumor suppressor and plays a pivotal role in apoptosis and cell cycle arrest [25-27]. In addition, various mutations of *p53 *were found to be associated with human cancers and the onset of MDR in a broad field of solid and hematological malignancies [28-34]. By performing the electrophoretic mobility shift assay (EMSA) and chromatin immunoprecipitation (ChIP) assays, we demonstrated that the *WTH3 *gene was a direct target of the p53 protein [24]. This relationship led us to evaluate the possible participation of *WTH3 *in promoting apoptosis via different approaches. Our findings suggested that over expression of *WTH3 *stimulated cell death [24]. As a result, we believed that this gene played an important role in MDR development.

To further understand *WTH3*'s involvement in MDR, we carried out shRNA knockdown experiments to see if reduced *WTH3 *expression would increase tolerance of host cells to the anti-cancer drug, Doxorubicin (Dox). In addition, considering the physical interaction of p53 and the sequences subjected to DNA methylation, and a current observation that treating MCF7/AdrR cells with 5-aza-2'-deoxycytidine (5-aza), a DNA methylation inhibitor, further elevated *p53 *transgene activity, which in turn increased endogenous *WTH3 *expression in host cells, we explored the possible interplay between epigenetic modification and the p53 transcription factor regarding their influence on *WTH3 *gene expression.

## Methods

### Cell Lines and Treatment

MCF7/AdrR and MCF7/WT were grown at 37°C with 5% CO_2 _in DMEM medium with 10% FCS, 100 μg/ml streptomycin and 100 U/ml penicillin. HEK293 (human primary embryonic kidney cells, ATCC.) and Hela cells were grown at 37°C with 5% CO_2 _in RPMI 1640 culture medium with 10% FCS, 100 μg/ml streptomycin and 100 U/ml penicillin. To determine the influence of DNA methylation on *p53 *activity as it pertains to endogenous *WTH3 *gene expression, MCF7/AdrR cells were treated with 5-aza at 50 μM (this high concentration used was due to that the MCF7/Adr cell line was extremely drug resistant, whose IC50 was 975 nM, while MCF7/WT's IC50 was 1.25 nM[10]) for 24 and 72 hours, while Hela cells were treated with 5-aza at 5 μM for 24 hours.

### Construction of Recombinant DNA

Detailed information about generating the full length *WTH3 *promoter in pGL3 to obtain the pGL/WTH3P construct and its deletion mutant with activity in pGL3 to generate the pGL/WTH3d3 construct were previously described [9,15]. Wild type *p53 *in pcDNA/P53 and the mutated *p53 *gene in pcDNA/P53R249S, which did not contain trans-element-activity, were gifts from Dr. Moll M. Ute.

### shRNA Knockdown

The *WTH3 *shRNA oligos, GATCCGTCAGGCAATAATTGGCATTGATTCAAGAGATCAATGCCAATTATTGCCTGACTTTTTTACGCGTG (sense) and AATTCACGCGTAAAAAAGTCAGGCAATAATTGGCATTGATCTCTTGAATCAATGCCAATTATTGCCTGACG (anti-sense) ending with *BamH *I and *EcoR *I restriction enzyme ends, were cloned into the pSIEN-RetroQ vector to obtain pSIEN-RetroQWTH3 according to the Clontech Knockout RNAi User Manual (PT3739-1). The infection procedure was performed based on the Clontech Retroviral Gene Transfer and Expression User Manual (PT3132-1). Briefly, pSIEN-RetroQWTH3 or pSIEN-RetroQ (negative control) along with the envelope vector, pAmpho were co-transfected into the GP2-293 packaging cell line via the phosphate calcium method. Viral supernatant was collected and centrifuged at 500 × g for 10 min to remove the cellular debris. The supernatant was then used to infect HEK293 cells. The cells with integrated *WTH3 *shRNA sequences were selected by adding 0.5 μg/ml puromycin into the medium for 2 weeks. The limiting dilution procedure was performed as previously described [9] to obtain individual HEK293 clones that were either integrated with pSIEN-RetroQWTH3 or pSIEN-RetroQ.

### SQRT-PCR

Total RNAs were isolated from cell lines treated with 5-aza, transfectants and the corresponding negative controls by the High Pure RNA Isolation Kit (Roche). SQRT-PCR was performed using the Titan One Tube RT-PCR system based on the manufacturer's protocol (Roche). The sense and anti-sense primers for *WTH3, MDR1 *and *GAPDH *were previously described [9]. The sense and anti-sense primers for *WTH3 *were 5'-GATGGAACAATCGGGCTTCG-3' and 5'-GCTGCTACACGTCGAAAGAGC-3'. The sense and anti-sense primers for *MDR1 *were 5'-CCTATCATTGCAATAGCAGG-3' and 5'-GTTCAAACTTCTGCTCCTGA-3'. The length of the *WTH3 *and *MDR1 *PCR product was 341 and 167 bps, respectively. The SQRT-PCR assay for each gene of interest was performed more than three times. The PCR and quantification of PCR products were performed as noted [9,10,15,23].

### MTT Assay

MTT assays were carried out as described [9,10]. Briefly, 2 × 10^3 ^cells/well were seeded in a 96-well plate and grown overnight. The cells were treated with serial concentrations of Dox (0 to 1 μg). In 6 days, the cells were then stained with 3- [4,5-dimethylthiazol-2-yl]-2,5-diphenyltetrazolium bromide (MTT). IC50 (IC50 values represent Dox concentrations that cause 50% cell death) was quantitatively measured at 595 nm by the program software, EZ-ED50 Version 1.1 (Perrella Scientific Inc,) in a micro-plate spectrophotometer (Benchmark Plus, BIO-RAD).

### *In Vitro *DNA Methylation

To methylate the CpG sites in pGL/WTH3P and pGL/WTH3d3 plasmids, 2 μg of each construct was incubated with 12 U CpG methylase *Sss *I and 640 μM of S-adenosylmethionine (SAM) (New England Biolab) at 37°C for 3 hr. The temperature was then increased to 65°C for 20 min to terminate the reactions. The methylated plasmids, pGL/WTH3P^m ^and pGL/WTH3Pd3^m^, were purified with the Qiaquick PCR Purification Kit (Qiagen). The success of *Sss *I methylation was verified by methylation sensitive restrictive enzymes, *Hha *I and *Hpa *II.

### Transient Transfection and Luciferase Assays

To determine whether the methylated *WTH3 *promoter influenced *p53 *transcriptional activity, pGL/WTH3P^m ^versus pGL/WTH3P and pGL/WTH3Pd3^m ^versus pGL/WTH3Pd3 were co-transfected with pcDNA/P53, pcDNA/P53R249S (negative control) or pcDNA/3.1 (negative control) into HEK293 cells. In brief, 0.2 μg of each construct were transfected along with 0.1 μg of pCMV/β-galactosidase when the cells (seeded onto 24-well plates) reached 50–70% confluence. After 24 hrs, luciferase and β-galactosidase activity was measured using the Luciferase Assay System and Beta-Glo™Assay System (Promega) according to the manufacturer's instruction. Luciferase activities of transfectants were compared after normalizing their β-galactosidase activities and protein concentrations.

## Results

### The HEK293 cells with *WTH3 *knockdown resulted in higher resistance to Dox and stimulated *MDR1 *gene expression

**Figure 1:**
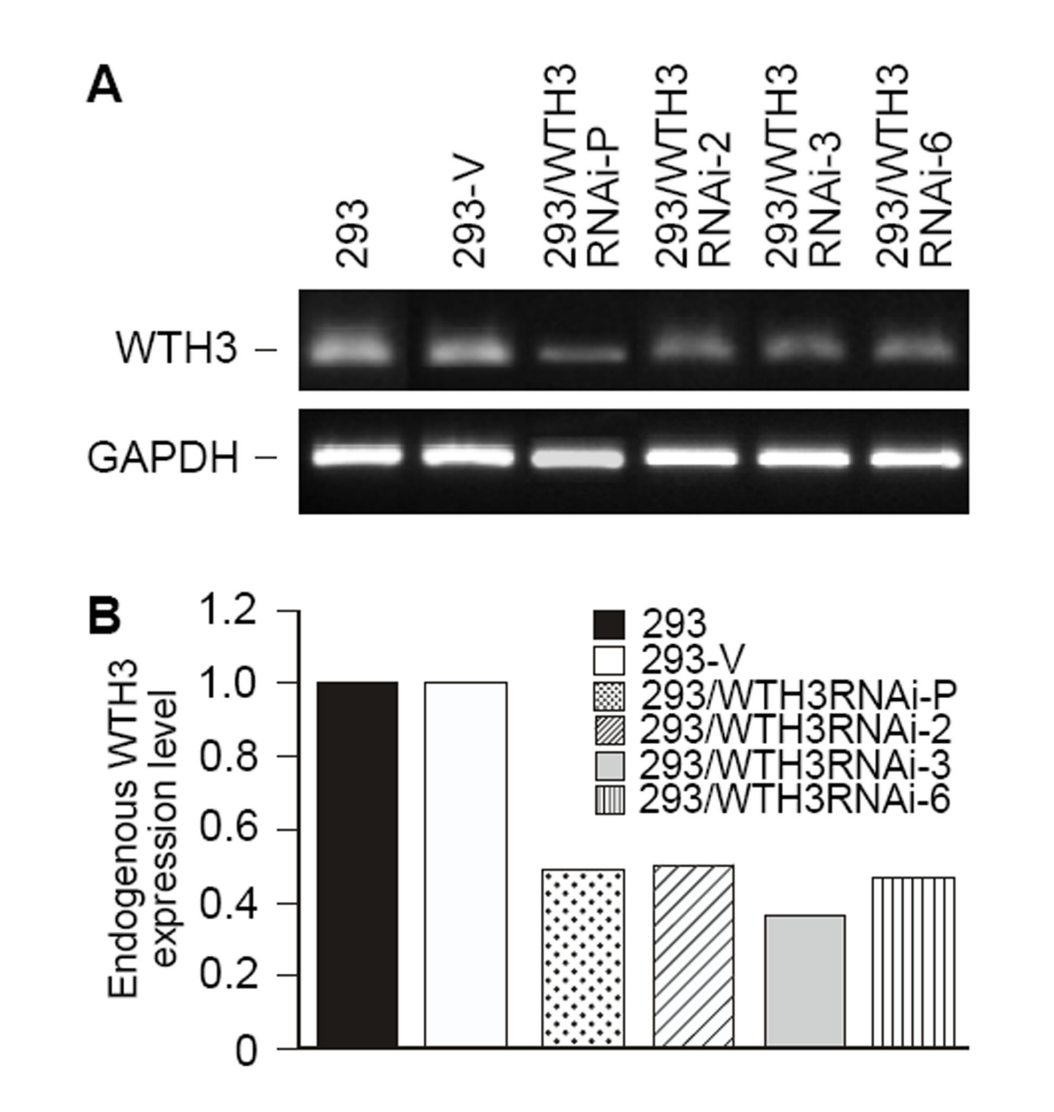
**SQRT-PCR results for *WTH3 *expression in the knockdown cells, population (293/WTH3RNAi-P), 3 cloned cell lines (293/WTH3RNAi-2, -3, and -6), and 293 control cells (293-V) that were integrated with the empty vector.** A. PCR products are displaced in an electrophoresis gel, while GAPDH served as positive control. B. Results of quantitative analysis for *WTH3 *expression in the cells presented in A. GAPDH served as quantitative reference.

**Figure 2:**
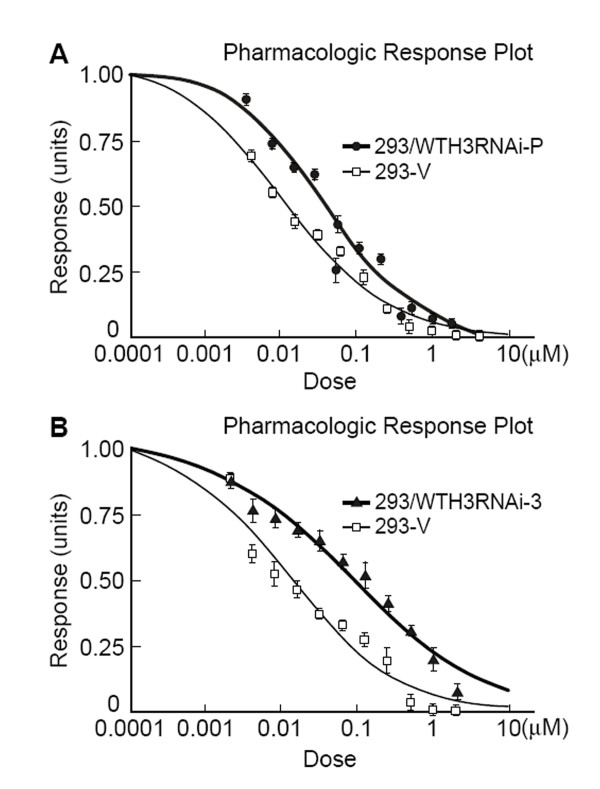
**MTT results obtained from the knockdown population and cloned cell lines, which were treated with Dox.** A. Pharmacologic responsive plot for the knockdown population, 293/WTH3RNAi-P, versus control 293-V. B. Pharmacologic responsive plot for the cloned knockdown cell line, 293/WTH3RNAi-3 versus control 293-V. C. IC50 values of WTH3 knockdown 293 cells versus 293-V control cells to Dox.

**Figure 3:**
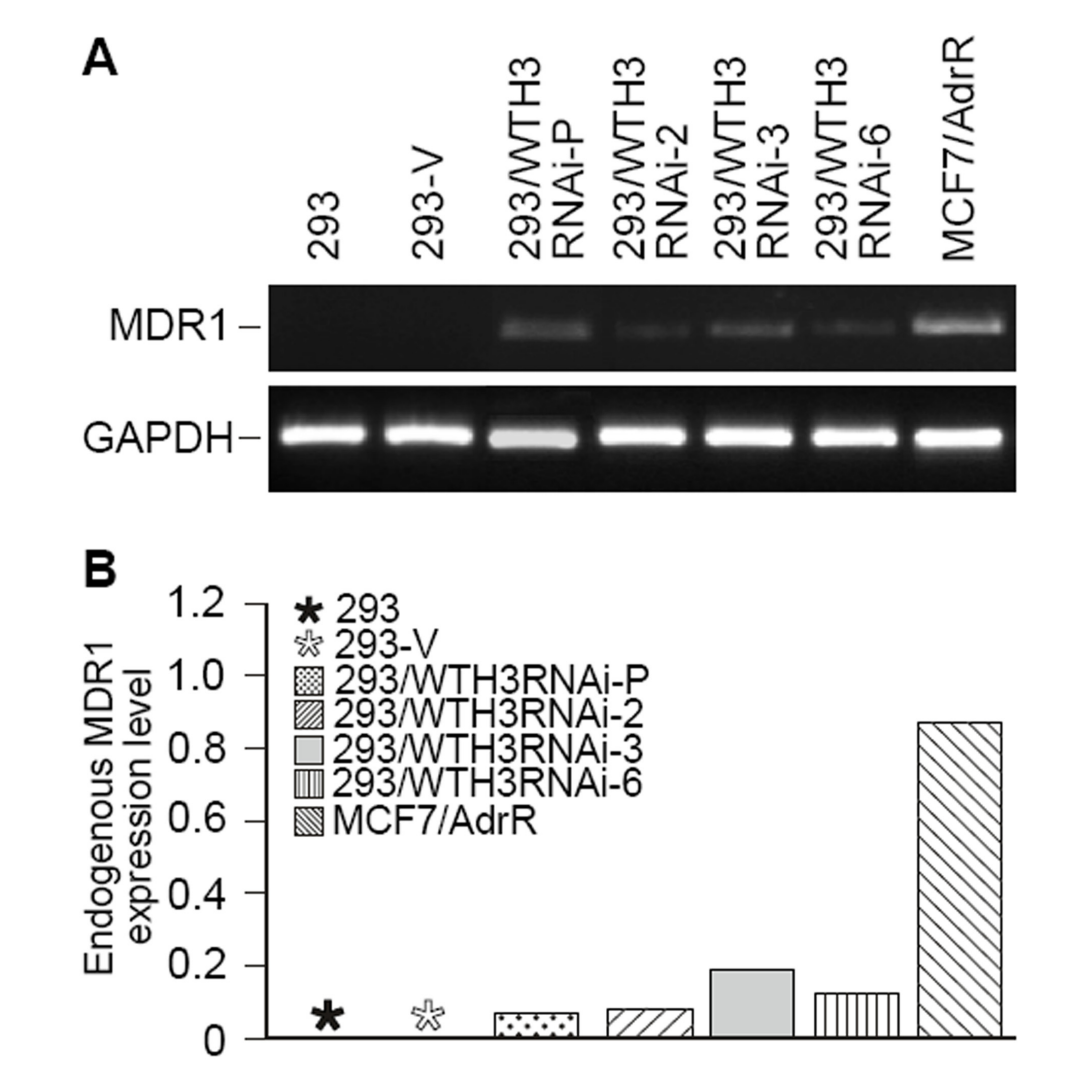
**SQRT-PCR results for *MDR1 *expression in population and cloned cell lines.** A. The *MDR1 *gene's PCR products in various cell lines are displaced in an electrophoresis gel, while *MDR1 *expression in MCF7/AdrR and GAPDH served as positive controls. B. Results of quantitative analysis for *MDR1 *expression in the cells presented in A. GAPDH served as quantitative reference.

Earlier studies suggested that *WTH3 *was involved in MDR development[9,23]. To further confirm this possibility, we decided to knockdown the *WTH3 *gene in HEK293 cells to see if reduced gene expression could increase the host cells' resistance to an anti-cancer drug, such as Dox. To do so, we employed the Clontech Knockout RNAi system to integrate a DNA sequence, which specifically interfered with the endogenous *WTH3 *transcripts, into the host cells' genome (see Materials and Methods for details). After antibiotic selection, RNAs were extracted from the cells infected with pSIEN-RetroQWTH3 or pSIEN-RetroQ (negative control), and quantified by SQRT-PCR. The results obtained from the three individual measurements showed that *WTH3 *transcripts in the cells infected with pSIEN-RetroQWTH3 (293/WTH3RNAi-P) were about 2 times lower than that in the control cells containing the empty vector (Fig. 1A, 1B). Therefore, the *WTH3 *gene was successfully knockdown in the HEK293 cell population. We then selected several individual cell clones via limiting dilution procedures. Consequently, we obtained 7 clones whose *WTH3 *expression was significantly lower due to the knockdown and 6 clones whose genome was integrated with the empty vector, but did not change *WTH3 *expression levels (Fig. 1A, 1B). The populations and three cloned cell lines, 293/WTH3RNAi-2, -3, -6, which were randomly picked, were used to perform MTT assays to obtain IC50s (the concentration of a drug that causes 50% cell death) to Dox. The results were an average of three individual experiments for each group. The IC50 value of the 293/WTH3RNAi-P cells was about 39 nM, while that of the control was about 13 nM. Clearly, the reduction of *WTH3 *gene expression made the host 3 times more resistance to Dox than its control, 293-V (Fig. 2A, 2B). Next, IC50s of the three cloned lines, 293/WTH3RNAi-2, -3 and -6 were also evaluated. The IC50 values to Dox were 44, 93, and 52 nM for 293/WTH3RNAi-2, -3 and -6, respectively, which were approximately 3.4, 7 and 4 times more resistance than the 293-V control (Fig. 2A, 2B, Table 1). Moreover, due to past findings that suggested a reverse correlation between *WTH3 *and *MDR1 *gene expression levels [23], we examined *MDR1 *gene expression levels in those cells by SQRT-PCR. As we expected, the results showed that the *MDR1 *gene was re-activated in the 293/WTH3RNAi-P, 293/WTH3RNAi-2, -3 and -6 cells, while *MDR1 *transcripts were undetectable in the corresponding control cells (Fig. 3A, 3B).

**Table 1:** IC50 values of 293 cells with WTH3 knockdown vs. control

**Cells**	**IC50 (nM)**	**Degree of resistance**
293-V	**13**	**1**
293/WTH3RNAi-P	**39**	**3**
293/WTH3RNAi-2	**44**	**3.4**
293/WTH3RNAi-3	**93**	**7**
293/WTH3RNAi-6	**52**	**4**

### 5-aza treatment promoted *p53*'s positive impact on *WTH3 *expression

**Figure 4:**
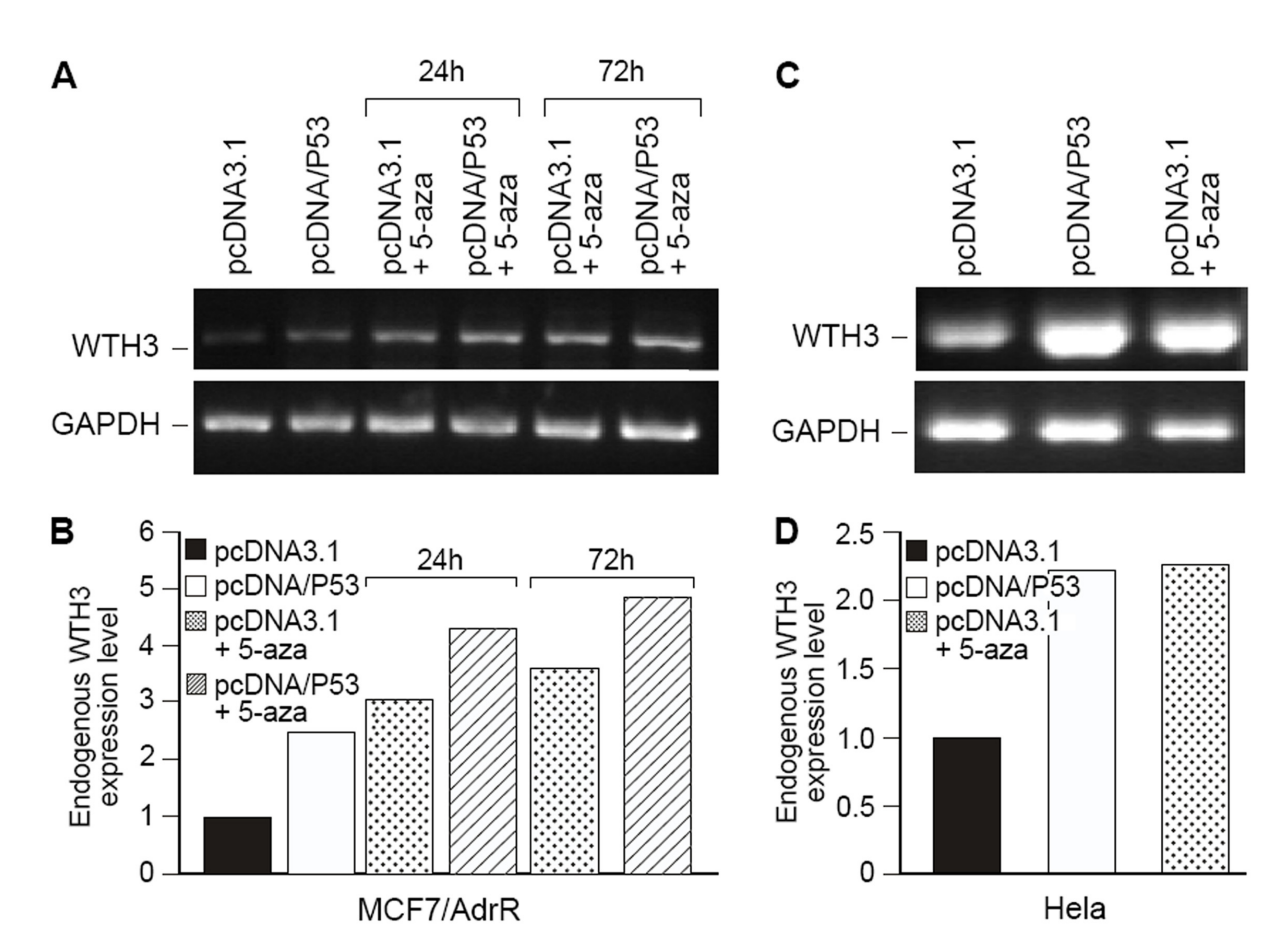
**SQRT-PCR results for endogenous *WTH3 *gene expression in MCF7/AdrR and Hela cells**. A. Electrophoresis gel display of the endogenous *WTH3 *gene expression in MCF7/AdrR cells that were transfected with pcDNA3.1 or *p53*, and transfected with pcDNA3.1 or *p53 *and treated with 5-aza for 24 or 72 hr. GAPDH served as positive control. B. Results of quantitative analysis for *MDR1 *expression in the cells presented in A. GAPDH served as quantitative reference.

Prior studies demonstrated that the CpG island found in the *WTH3 *gene promoter was targeted by DNA methylation in MCF7/AdrR cells [15,23]. It was also discovered that this island contained p53M, which was directly bound by the p53 protein, a trans-element for activating *WTH3 *gene expression [24]. Based on these facts we speculated that DNA methylation could play an antagonistic role influencing the *p53 *transcription factor. To test this hypothesis, we transfected the pcDNA/P53 or pcDNA3.1 (negative control) construct into MCF7/AdrR cells (whose CpG island in the *WTH3 *promoter was methylated and *p53 *defective due to a mini-deletion) [32], and then treated the transfectants with 5-aza to see if the *p53 *transgene preferentially activated the *WTH3 *gene expression, but not in the untreated cells. After 24 and 72 hours of treatment, endogenous *WTH3 *transcript levels were evaluated by SQRT-PCR. We found that the *p53 *transgene's positive effect on the *WTH3 *gene expression of the cells treated with 5-aza was about 2 times stronger than that in the control cells who either contained the transgene or the empty vector, but were not treated with 5-aza (Fig. 4A, 4B). This suggested that DNA methylation could negatively affect p53 transactivity. In addition, as we expected, the 5-aza treat alone increased *WTH3 *transcript. Considering that Hela cells express a relatively low level of *WTH3 *and a relatively high IC50 value to Dox (data not shown), we introduced *p53 *transgene into those cells or treated them with 5-aza. It was observed that the *p53 *transgene and 5-aza positively affected the *WTH3 *expression of the cells compared to the untreated cells (Fig. 4C, 4D). These results further indicated that epigenetic modification and *p53 *regulated the *WTH3 *gene. To verify if DNA methylation could negatively affect p53 transactivity, the *in vitro *DNA methylation approach was employed to modify full length and deleted *WTH3 *promoters to analyzed *p53*'s influence.

### *P53 *failed to activate the methylated *WTH3 *promoter

**Figure 5:**
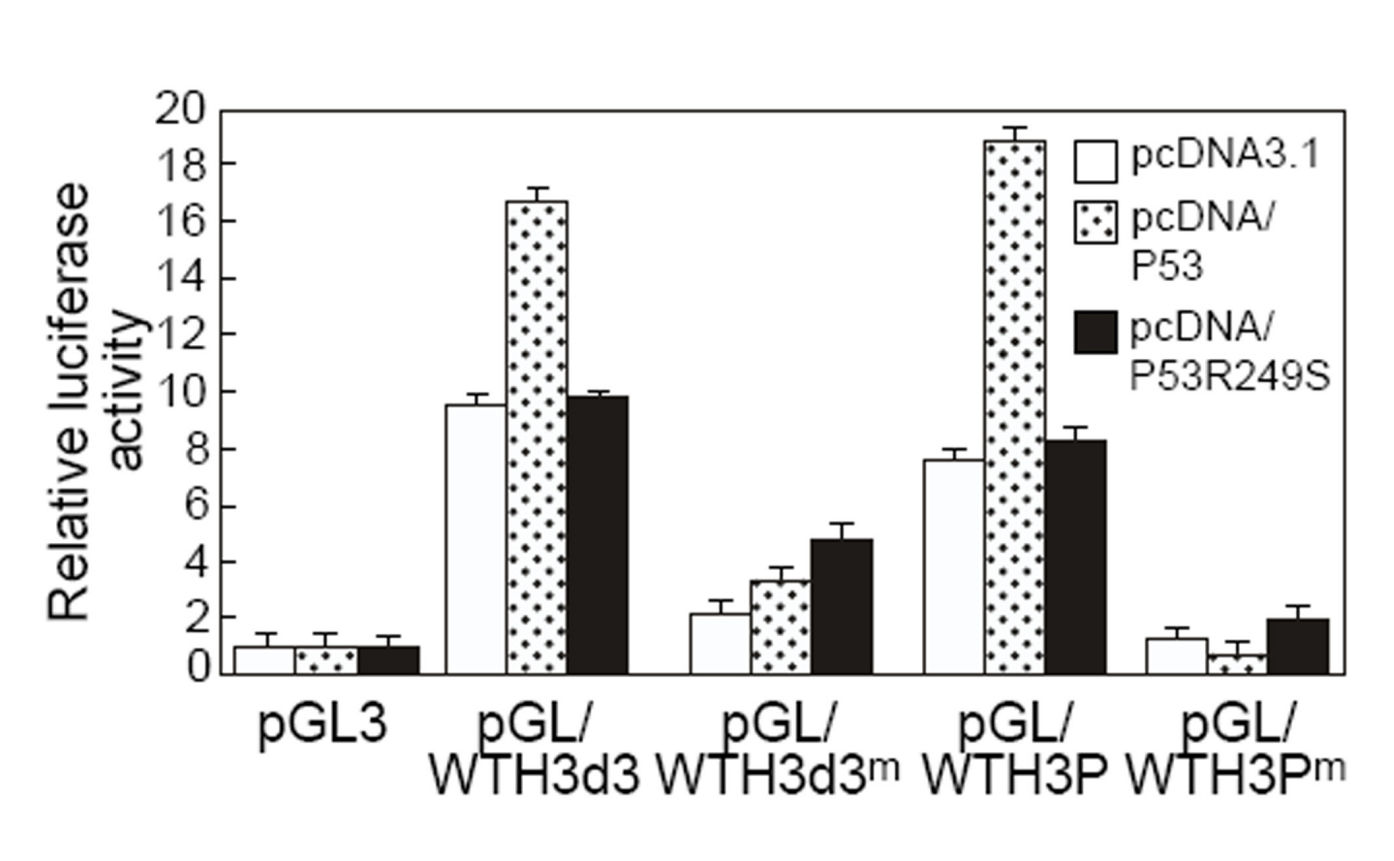
Luciferase activity driven by the methylated and non-methylated full length and deleted *WTH3 *promoters, pGL/WTH3P^m^, pGL/WTH3P, pGL/WTH3Pd3^m ^and pGL/WTH3Pd3 when they were co-transfected with pcDNA/P53, pcDNA/P53R249S, or pcDNA3.1 into HEK293 cells.

To methylate full length and deleted *WTH3 *promoters, pGL/WTH3P and pGL/WTH3d3 were incubated with *Sss *I methylases and SAM. The resulting construct, pGL/WTH3P^m ^and pGL/WTH3d3^m^, their non-methylated controls, as well pCMV/β-galactosidase (transfection efficiency control) were then introduced into HEK293 cells. As we assumed, luciferase activities driven by both methylated promoters were 5 times lower than the corresponding controls (Fig. 5). Clearly, DNA methylation inhibited the promoters' function. We then co-transfected pGL/WTH3P^m ^versus pGL/WTH3P and pGL/WTH3d3^m ^versus pGL/WTH3d3 with pcDNA/P53, or pcDNA/P53R249S (negative control), or pcDNA3.1 (negative control) into HEK293 cells to see if DNA methylation could antagonize p53 transactivity. The results showed that the *p53 *transgene only activated the non-methylated WTH3P and WTH3d3 promoters but had no effect on their methylated counterparts (Fig. 5). However, mutated p53, p53R249S, was unable to influence non-methylated WTH3P and WTH3d3 promoters. These findings indicated that there was interplay between DNA methylation and the p53 transcription factor regarding *WTH3 *gene expression regulation.

## Discussion

Earlier studies suggested that *WTH3 *could be an important gene involved in the cellular MDR phenotype development. This idea was based on three observations, 1) its expression was reduced in cells exhibiting drug resistant traits [9,15,23]; 2) its restoration in MDR cell lines not only increased sensitivity to a variety of drugs, but also decreased endogenous *MDR1 *gene expression [9,15,23]; and 3) it was a direct target of *p53 *whose role during the onset of MDR has been well documented [26,30,32,35-38]. To further verify *WTH3*'s importance related to drug resistance, we recently employed the small hairpin RNA interference technique to permanently reduce its expression in non-MDR HEK293 cells to see if this could increase the host's tolerance to Dox. The reason for utilizing HEK293 was that it expressed normal amounts of the *WTH3 *gene, but undetectable *MDR1 *RNA, and was quite sensitive to Dox. The knockdown procedure was considered successful because *WTH3 *gene expression in the population and cloned host lines was 2 or more times below the original level. After measuring their IC50 values to Dox we found that all those cells with the *WTH3 *knockdown were 2 to 4 times more resistant to Dox than their controls. In addition, the knockdown was accompanied by significant *MDR1 *gene re-activation. These results were consistent with previous observations and supported the notion that there was a direct link between *WTH3 *gene function and MDR development. This relationship would be even more obvious if the *WTH3 *gene was completely knocked out by a traditional targeting knockout approach. This prediction is based on the fact that *WTH3 *gene expression was much lower (about 10 times) in MCF7/AdrR than in MCF7/WT cells, the former line processing a much stronger (about 100 times) MDR phenotype than the later one.

Considering that the *WTH3 *gene could play an important role in regards to the on set of MDR, we were interested in understanding the detailed mechanisms by which its transcription was regulated by DNA methylation and the p53 transcription factor. In the past, we presented data that the *WTH3 *gene promoter was hypermethylated not only in MCF7/AdrR but also drug resistant primary breast cancer epithelial cells [15,23] and was physically targeted by p53 proteins [24]. We also found that the p53-binding site, p53M, in the gene promoter resided in a CpG island that was subjected to epigenetic modification. This information indicated that DNA methylation, whose role is to repress gene expression [39] could have a direct impact on *p53 *activated *WTH3 *expression. To test this hypothesis, two approaches were employed. The first included evaluating the *p53 *transgene's influence on *WTH3 *expression in MCF7/AdrR cells that were treated with 5-aza. The second was to study *WTH3 *promoter activity that was modified by methylation. The resulting information pointed out that DNA methylation significantly diminished promoter activity by at least one mechanism, by antagonizing *p53 *transactivity. Since, DNA methylation usually attracts methyl-binding-proteins (MBPs) and other epigenetic modification factors (EMFs), one could image that the methylated *WTH3 *promoter was bound by some MBPs and EMFs, which might change the chromatin structures and prevent p53 from recognizing its targeting structures.

## Conclusion

Taken together, our studies provided solid evidence supporting the important role played by the *WTH3 *gene in MDR development and uncovered one of the mechanisms regulating its expression. Therefore, restoring or increasing this gene's activity could be another valuable strategy for easing MDR encountered during cancer chemotherapy. This could be achieved by introducing demethylation reagents if attenuated *WTH3 *gene activity in a patient was caused by DNA methylation.

## Competing interests

The authors declare that they have no competing interests.

## Authors' contributions

KT participated experiments designing and carried out shRNA Knockdown, DNA methylation and luciferase assay. YW participated cell culture and molecular cloning. YH carried out MTT assay. BS did the PCR and SQRT-PCR. YL carried out shRNA construction. HX conceived of the study, participated in its design and coordination, and drafted the manuscript. All authors read and approved the final manuscript.

## Pre-publication history

The pre-publication history for this paper can be accessed here:


